# The Presidency

**Published:** 1885-01

**Authors:** Geo. E. Blackham


### THE PRESIDENCY.

GEO. E. BLACKHAM, M. D.

The privilege of electing our rulers is, no doubt, one of the chief blessings for which we, as a people, ought to be thankful.

It is a good thing to change our rulers from time to time, and yet, in view of the intense bitterness of the late campaign and of the disturbance of business always caused by a presidential election, it seems evident that we may have too much of a good thing.

The term of office of the President of the United States is so short, only four years, that it scarcely suffices for our chief magistrate to become familiar with the grave and complex duties of his high office, and offers no opportunity for the proper development of a statesmanlike policy on the part of the incumbent.

By the time he has become reasonably familiar with the mere routine of official life and has succeeded in surrounding himself with officials in harmony with his views, he is so near the end of his term that he must give up, for lack of time, all hopes of carrying into execution the plans he has formed, or must begin to scheme and plot for a re-election.

The ablest statesman might well despair of doing much good while hampered with such restrictions, while a man of less ability might, if the term of office were longer, grow up to his position, and, with opportunity for deliberate action, develope a policy that should be of permanent benefit to his country.

Thus much as to the evil effects of short presidential terms upon the usefulness of the occupants of the highest office in the gift of the American people.

The direct effect upon the people is not less marked. The frequent recurrence of presidential elections and the liability to a radical change of policy once in four years, tend to destroy that confidence which is founded upon a sense of the stability of our institutions; and the frequent recurrence of presidential campaigns with their attendant evils of partizan bitterness, emotional excitement, and the partial diversion of the attention of the citizens from their legitimate business to the childish excitements and display of campaign organizations, parades, &c., &c., tend to break up the orderly course of business life and to subject the people to a sort of remittent political fever, the exacerbations of which follow one another so rapidly that the National head has not time to cool nor the National pulse to quiet down after one attack before they begin to feel the prodromic symptoms of the next.

Of course, if an ideal state of society existed, if citizens could come together and calmly discuss the policy of the government without excitement or prejudice and then quietly select the person deemed best qualified to carry out the views deemed, by the majority, best for the interests of the whole country, and if we could have presidents free from the weaknesses of humanity, who would or could do right for right's sake and without thought of the influence of their action upon their personal ambitions or could be sure that this course would assure their continuance in power as long as they followed it, our frequently recurring elections would be deprived of their evil influences and become the means of making our system a self-adjusting one, flexible enough to adapt itself to changing circumstances, and yet stable enough to inspire confidence.

But, unfortunately, no such Utopian state of affairs exists and, in spite of all the spread-eagle oratory of the Fourth of July speakers, can hardly be looked for much in advance of the millenium.

We must take things as we find them and do the best we can with them, and if we find that certain parts of our governmental system fail to work well in practice, however perfect they may be in theory, we must amend them.

For the evils resulting from short term presidencies with their hampering of the President in the matter of the policy of his administration, the temptation to use the power of his first term as a means to securing a second, with their disturbing effect upon the emotions, temper and business prosperity of the people at large, two remedies suggest themselves:

1. The adoption of some modification of the British parliamentary system by which a party or an administration holds power just so long as it retains the confidence and support of the people; or 2nd, the lengthening of the presidential term and making the president ineligible to re-election.

While the first plan, i. e. the adoption of the British parliamentary system, is doubtless the fairest and most satisfactory in theory, there are certain difficulties in the way of its adoption, and, in addition, it is more than doubtful if our people could be brought to accept an innovation that smacked of monarchy, notwithstanding the fact that it gives the voting population more absolute control over the personel and policy of the government than any other that has, as yet, been devised.

The only alternative, then, is to lengthen out the term of the presidential tenure of office, and to make the person who has once *been elected* president ineligible as a candidate for ever after.

I would then suggest the increase of the presidential term from four to ten years, the president to be ineligible for re-election, to be retired at the end of his service on a liberal pension and with a life position as Senator at Large.

I would couple with this the total abolition of the Vice Presidency, as the office is an utterly superfluous one except in case of the death of tne President.

In support of these propositions may be urged—

1. To increase the presidential term to ten years is to give the president time to familiarize himself with the duties and opportunities of his position and to utilize that knowledge with deliberation before having to make way for his successor or to turn his attention to his own plans for re-election.

2. To make the president ineligible for re-election is to relieve him in great measure of the temptations which every man must feel, to prostitute his high office for the furtherance of his personal ambition.

3. To retire him with a liberal pension and a life position as U. S. Senator at Large, is to relieve him from temptation to use his position for his pecuniary benefit, to provide him with honorable and dignified occupation for the remainder of his days, and to secure to the people the fruits of the experience he has gained while occupying the highest position in their gift.

4. The abolition of the Vice Presidency is advocated because the office is an anomaly and a superfluity, and it only serves to provide for the presidential succession in case of the death of the President.

But, in so doing, it actually provides that the highest office in the Nation may be held by a person not chosen to that office by the people. It would seem to accord better with the spirit of our institutions to abolish the office of Vice President, to allow the Senate to elect its own presiding officer, and, in case of the death of a President during his term of office, to allow his Secretary of State to act as President *pro tem.* until Congress could be called together (if not in session) and a new President elected by the people for the unexpired portion of the late President's term (or better, for a full term of ten years).

If with these changes could be coupled the abolition of the electoral college system, which has been totally diverted from its original purposes and the substitution of the direct election of the President by a majority, or plurality, of the total popular vote of the Nation, it seems to me that we should secure advantages well worth the trouble of making the change.

I would not, however, be understood as offering this scheme as a complete panacea for all the ills resulting from our present system, nor even as the best possible system that can be devised, but as an earnest and serious contribution to the solution of a problem that has presented itself to the minds of many American citizens with whom country is greater than party.

HINTS FOR MEDICAL STUDENTS.—According to the *Boston Medical and Surgical Journal*, the following was found posted on the bulletin board of the Harvard Medical School after the late examinations: "Dr. Edes wishes to call the attention of the graduating class to the following facts, which may be important to them and their patients. The doses referred to are NOT all from books which failed to receive 50 p. c. The following are too large does: Half a grain of morphia, ten drops of Fowler's solution, to begin with; 10 c. c. of fl. ext. of conium; calomel, 7 grains; $\frac{1}{8}$ or $\frac{1}{4}$ grain of sulphate of atropia; 1 gram of sulphate of copper; half a gram of aloes every night in habitual constipation. The following would make inconveniently large pills: One gram of pil. aloes et myrrh (no such pill made is offinal); 390 grams of of sulphate of quinine and 130 grams of confection of roses made into six pills; 7 grams of calomel and 7 grams of calomel and six centigrams of blue mass. Pepsine and bismuth should not be given together. A teaspoonful of limewater in a tumbler of milk does neither good nor harm. Calomel should not be used as an habitual cathartic (as was recommeded in a good many books). Mercury alone cannot conveniently be made an ingredient of an extemporaneous pill. Blue pill does not contain calomel. An apothecary cannot reasonably be expected to go out and kill a pig to make fresh pepsine for each prescription that comes in."

